# Objective Understanding of Front-of-Package Nutrition Labels: An International Comparative Experimental Study across 12 Countries

**DOI:** 10.3390/nu10101542

**Published:** 2018-10-18

**Authors:** Manon Egnell, Zenobia Talati, Serge Hercberg, Simone Pettigrew, Chantal Julia

**Affiliations:** 1 Sorbonne Paris Cité Epidemiology and Statistics Research Center (CRESS), U1153 Inserm, U1125 Inra, Cnam, Paris 13 University, Nutritional Epidemiology Research Team (EREN), 93000 Bobigny, France; s.hercberg@eren.smbh.univ-paris13.fr (S.H.); c.julia@eren.smbh.univ-paris13.fr (C.J.); 2 School of Psychology, Curtin University, Kent St, Bentley, WA 6102, Australia; zenobia.talati@curtin.edu.au (Z.T.); simone.pettigrew@curtin.edu.au (S.P.); 3 Public health department, Avicenne Hospital, AP-HP, 93000 Bobigny, France

**Keywords:** nutritional labelling, international comparison, comprehension

## Abstract

Front-of-Package labels (FoPLs) are efficient tools for increasing consumers’ awareness of foods’ nutritional quality and encouraging healthier choices. A label’s design is likely to influence its effectiveness; however, few studies have compared the ability of different FoPLs to facilitate a consumer understanding of foods’ nutritional quality, especially across sociocultural contexts. This study aimed to assess consumers’ ability to understand five FoPLs [Health Star Rating system (HSR), Multiple Traffic Lights (MTL), Nutri-Score, Reference Intakes (RIs), and Warning symbol] in 12 different countries. In 2018, approximately 1000 participants per country were recruited and asked to rank three sets of label-free products (one set of three pizzas, one set of three cakes, and one set of three breakfast cereals) according to their nutritional quality, via an online survey. Participants were subsequently randomised to one of five FoPL conditions and were again asked to rank the same sets of products, this time with a FoPL displayed on pack. Changes in a participants’ ability to correctly rank products across the two tasks were assessed by FoPL using ordinal logistic regression. In all 12 countries and for all three food categories, the Nutri-Score performed best, followed by the MTL, HSR, Warning symbol, and RIs.

## 1. Introduction

In 2016, non-communicable diseases (e.g., cardiovascular disease, cancer, obesity, and type 2 diabetes) were responsible for 39.5 million deaths worldwide [1]. For these diseases, nutrition-related behaviours are recognised as some of the main risk factors and are considered key elements in public health policies, as they represent modifiable determinants of health that can be addressed through primary prevention interventions [2,3,4,5,6]. Therefore, various strategies and public policies have been introduced worldwide to improve people’s diets [7,8,9,10,11]. Among them, the provision of nutrition information via front-of-pack labels (FoPLs) has been attracting growing attention from public health authorities. As FoPLs provide information on the nutritional content (or quality) of pre-packaged food products, they can help consumers to make heathier food choices at the point of purchase [4,10,12]. Moreover, FoPLs are postulated to encourage food manufacturers to reformulate to increase the healthfulness of their products to improve the FoPLs shown on the foods [13,14]. Due to these individual and market-level considerations, simulation studies suggest that the adoption of FoP nutrition labelling constitutes a cost-effective means of achieving health benefits [15,16]. 

For a FoPL to be useful in purchasing situations, consumers need first to understand the information they provide [17]. Understanding can be distinguished as either subjective or objective understanding. Subjective understanding refers to the meaning attached by consumers to the label information and the extent to which they believe they have understood this information, while objective understanding is defined as the consumer’s capacity to interpret the information conveyed by the FoPL as intended by its designers [17]. As such, a subjective understanding is usually captured by a self-administered questionnaire including a self-report by participants on the extent to which they believe they understand the information conveyed by a FoPL. Objective understanding, on the other hand, is captured by requiring participants to complete a task in which understanding is tested, such as ranking or selection tasks with visuals of food products displaying FoPLs. Objective understanding is influenced by a number of factors, both at the individual level (e.g., interest in and/or knowledge about nutrition, sociodemographic characteristics) and at the FoPL level (e.g., graphical design) [17]. Over the last decade, a number of different types of label designs has been developed, including nutrient-specific labels that display information on the content of a given nutrient and summary labels that provide an assessment of the overall nutritional quality of a given food product. Nutrient-specific labels can be divided into three categories: (i) numeric-only, such as the Reference Intakes (RIs) developed in 2006 and applied internationally by the food industry [18]; (ii) colour-coded labels, such as the Multiple Traffic Lights (MTL) label that was first implemented in the United Kingdom (UK) in 2005 (with each colour associated with the nutrient amount: red for a high amount, amber for a moderate amount, and green for a low amount) [19]; and (iii) warning labels, such as the Warning symbol (first implemented in 2016 in Chile [20]) that advises when the level of a given nutrient exceeds what is considered a healthy amount. Summary FoPLs can be categorised as (i) scale-based graded labels indicating the overall nutritional quality of the product, such as the Nutri-Score adopted in France in 2017 [21] and the Health Star Rating (HSR) system that first appeared on food packages in Australia in 2014 [22]; and (ii) endorsement symbols applied only to healthier products in a given food category and based on pre-set limits regarding the level of certain nutrients. Examples include the Choices label introduced in the 2000s in the Netherlands [23] and the Green Keyhole symbol introduced in the 1980s in Sweden and later in Denmark [24]. Except for nutrient-specific numeric FoPLs, which are purely informative, all other labels entail some level of interpretation of nutritional content through the use of colours, graphics, and/or textual elements and can be considered as interpretive labels. 

Literature reviews have concluded that FoPLs are generally favourably perceived and can increase consumers’ awareness of the healthiness of various food products [25,26,27]. Moreover, interpretive labels tend to be better understood by consumers than purely informative labels [28]. In recent years, there has been a steep increase in the number of studies comparing the effectiveness of various FoPLs [29,30,31,32,33,34,35,36,37,38,39,40]; however, the number of FoPLs compared in each study is typically small and more recent models (such as warning labels and summary graded FoPLs) are understudied. A growing number of countries are considering introducing FoPLs as a national public health tool, and some studies have revealed differences in consumer understanding and the effectiveness of FoPL formats across countries [40,41]. However, studies comparing different FoPLs across diverse cultural contexts are scarce.

To address this research gap, an international comparative study with an experimental design was conducted by two research teams to assess the effectiveness of various FoPLs across 12 countries. The FOP-ICE (Front-Of-Pack International Comparative Experimental) study investigated various aspects of consumer’s reactions to FoPLs, including attitudes, understanding, and impact on food choice. The present analysis focuses on consumers’ objective understanding of five FoPLs currently in use around the world (including nutrient-specific and summary labels: HSR, MTL, Nutri-Score, RIs, and Warning symbol) using a randomised experimental design. 

## 2. Methods

### 2.1. Participants

From April to July 2018, 12,015 participants were recruited in Argentina, Australia, Bulgaria, Canada, Denmark, France, Germany, Mexico, Singapore, Spain, the UK, and the United States of America (USA). In each country, recruitment was carried out through the same ISO-accredited international web panel provider (PureProfile) using quota sampling accounting for age (one-third of recruited participants in each of the following age categories: 18–30 years, 31–50 years, over 51 years), sex (50% women), and socioeconomic status (one-third of recruited participants in each of the following household income levels: low, medium, and high), to ensure equal coverage of the major population groups. Income brackets were calculated by estimating the median household income within each country and then creating a bracket of ±33% around this median, corresponding to the medium income band. Incomes below or above were considered as low- or high-income bands, respectively. To increase the ecological validity of the study, individuals who reported never or rarely purchasing at least two of the three food product categories tested in the study (pizzas, cakes, and breakfast cereals) were deemed ineligible to participate, because they would be unlikely to make these purchase decisions in real life. 

The protocol of the present study was approved by the Institutional Review Board of the French Institute for Health and Medical Research (IRB Inserm n°17-404) and the Curtin University Human Research Ethics Committee (approval reference: HRE2017-0760).

### 2.2. Design and Stimuli

**Figure 1:**
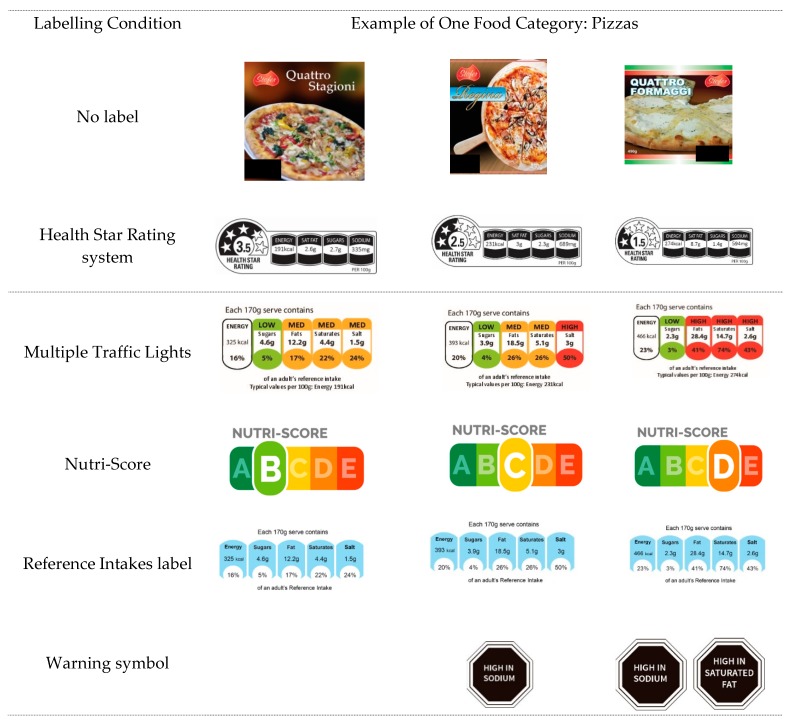
Example of the set of pizzas used for ranking tasks in the present study, with the associated FoPLs. The black rectangle at the bottom corner of the figure corresponds to the placement of the label.

Three food categories were selected for stimuli development according to two main criteria: (i) high variability in nutritional quality within the category and (ii) consumed in all 12 countries included in the study. Mock packages representing a fictional brand (“Stofer”) were used as stimuli to prevent other factors from interfering with product evaluation (e.g., familiarity, loyalty, and habit). The mock packages were created to resemble real food products, and a zoom function was developed to allow participants to enlarge any area of the package, including the FoPL. Within each food category, a set of three products with distinct nutritional profiles (lower, intermediate, and higher nutritional quality) was created to allow ranking, and the same food products were used across the different FoPL conditions. No other nutritional information or quality indicators (e.g., organic certification) appeared on the mock packages, so as not to influence participants’ perceptions of the products. All FoPL variants appeared in the same place on a given food product, and covered roughly the same surface area on the package. An example of the set of pizzas used in the study with the five corresponding FoPLs tested is shown in Figure 1; the two other sets of cakes and breakfast cereals are shown in Figures S1 and S2.

### 2.3. Procedure

Participants were invited to complete an online survey hosted by an international web panel provider. For each country, the online survey was translated into English for Australia, Canada, Singapore, the UK, and the USA; Spanish for Argentina, Mexico, and Spain; German for Germany; French for France; and Bulgarian for Bulgaria. Eligible participants were asked to provide information on their sex, age, income, household composition, educational level, involvement in grocery shopping, and self-estimated level of nutrition knowledge and diet quality. Following the socio-demographic, lifestyle, and nutrition-related questions, participants were presented with the initial task that asked them to rank the three sets of three label-free products (one set of three pizzas, one set of three cakes, and one set of three breakfast cereals) according to their nutritional quality. For each product, participants could choose from the following options: “1—Highest nutritional quality”, “2—Medium nutritional quality”, and “3—Lowest nutritional quality” (an “I don’t know” option was also included). Participants were subsequently randomised to one of the five FoPL conditions (HSR, MTL, Nutri-Score, RIs, and Warning symbol) and asked to repeat the same ranking task, this time with one of the five FoPLs displayed on the mock packages, according to the randomisation arm. Participants were not aware that they would be seeing the products twice, or that a FoPL would be present on the second viewing. Any potential presentation order effects were controlled for by randomising the order in which the products and the categories appeared on the screen. Participants’ objective understanding of a FoPL was assessed by comparing their ranking task results between the no label and FoPL conditions. It estimated the ability of individuals to use information conveyed by the FoPL to correctly rank products according to their nutritional quality compared to the no label condition. At the end of the survey, participants were asked whether they recalled seeing the FoPL to which they were exposed. The study protocol has been described in detail elsewhere: http://www.ANZCTR.org.au/ACTRN12618001221246.aspx. 

### 2.4. Statistical Analysis

Sociodemographic and lifestyle characteristics and FoPL recall were summarised by country and for the full sample. If a participant reported never purchasing products from a particular food category, his/her response to the corresponding ranking task was excluded. Next, for each participant and food category, the number of correct responses was calculated for the no label and the FoPL tasks. Ranking was considered correct if all the three products were ranked in the expected order and incorrect if any of the products were ranked out of order. The change in the number of correct responses across the three food categories from the no label to the FoPL condition was computed for each participant and expressed as a percentage.

The main outcome variable was the change in the number of correct responses between the FoPL and no label conditions. This was computed for each food category, leading to a category score of between −1 (deterioration) and +1 (improvement), with 0 denoting no change. Participants’ scores were then summed across the three categories, resulting in a final global score ranging from −3 to +3. Given the limited number of response options for the outcome variable, multivariable ordinal logistic regression was used to evaluate the association of FoPLs with change in the ability to correctly rank products from the no label to the FoPL conditions. Given the previous lower performance of the RIs reported in the literature, this FoPL was used as the reference category in the ordinal logistic regression models. Individual characteristics taken into account as covariates included sex, age, educational level, household income, involvement in grocery shopping, and self-estimated nutritional knowledge and diet quality. Variables displaying statistical significance at the *p*-value < 0.25 level in bivariate models were included in the multivariable model. For analyses including the full sample, the country was also included as a covariate. Sensitivity analyses were performed following exclusion of participants who did not recall seeing the FoPL during the survey. A false discovery rate approach was used to take into account multiple comparisons. A *p*-value below 0.05 was considered statistically significant. Statistical analyses were carried out using the full sample and by country, for all food categories combined and by individual food category, using SAS Software (version 9.3, SAS Institute Inc., Cary, NC, USA).

## 3. Results

Between April and July 2018, 12,015 participants responded to the online survey and were included in analyses (Table 1). The average time spent by the participants on the online questionnaire was 10.7 min, resulting in 0.45 min per item. Overall, 33.8% of participants had an undergraduate degree, 74.5% were responsible for grocery shopping, 64.9% reported having a mostly healthy diet, and 60.8% reported being somewhat knowledgeable about nutrition. Across the whole sample, 62.2% of participants recalled seeing the FoPL to which they were randomised. The two FoPLs with the lowest rate of recall were the Warning symbol (48.4%) and HSR (56.5%). 

**Figure 2:**
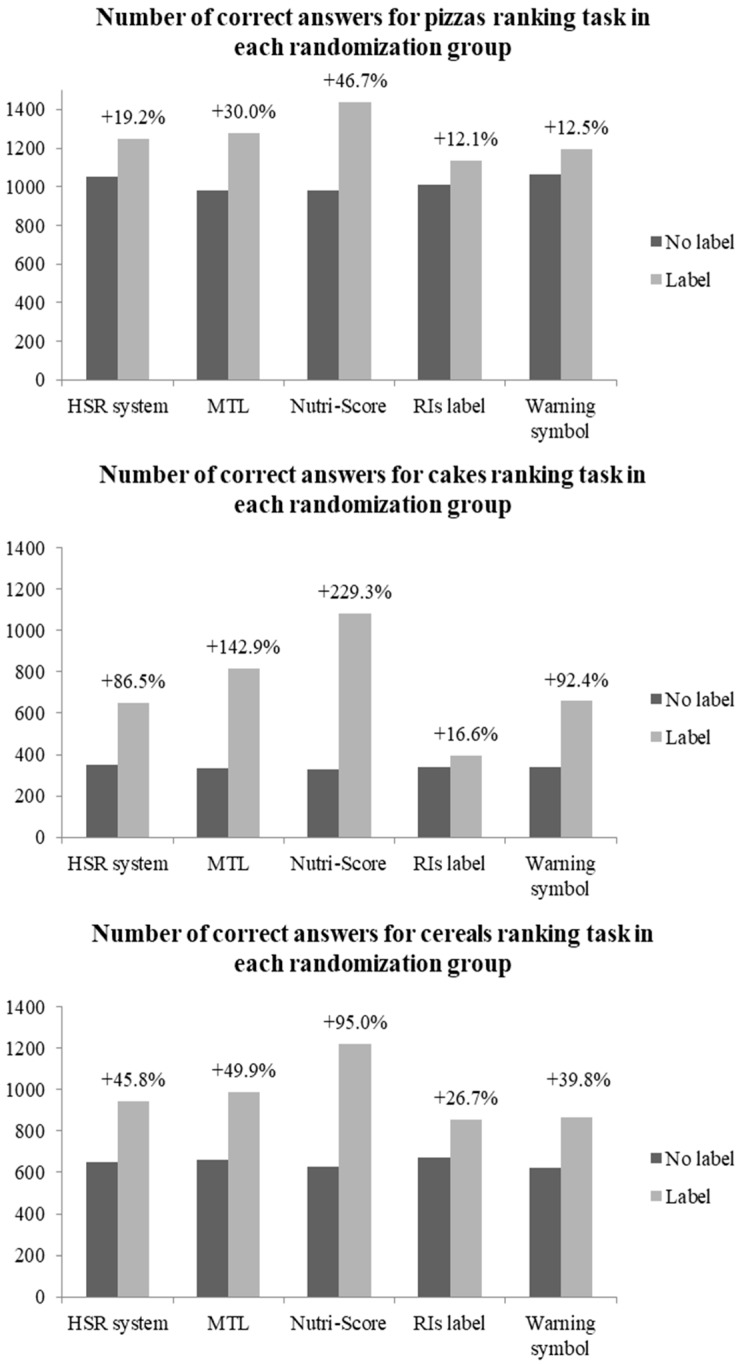
Number of correct answers for the total sample with the change compared to no label, by FoPL and food category.

The number of correct responses by food category by FoPL is presented in Figure 2. All five FoPLs improved the number of correct responses in the ranking task compared with the no label situation. However, large disparities were observed among the labels. For all countries combined, the Nutri-Score elicited the largest increase in the number of correct responses compared with the no label situation (+47% for pizzas, +229% for cakes, and +95% for breakfast cereals). This was followed by the MTL (+30% for pizzas, +143% for cakes, and +50% for breakfast cereals), the HSR (+19% for pizzas, +87% for cakes, and +46% for breakfast cereals), and the Warning symbol (+13% for pizzas, +92% for cakes, and +40% for breakfast cereals). Finally, the RIs elicited the smallest increase in the number of correct responses (+12% for pizzas, +17% for cakes, and +27% for breakfast cereals). Overall, similar patterns were observed in each country (data not shown). 

Associations between FoPLs and change in participants’ ability to correctly rank products according to their nutritional quality are displayed in Table 2. In the full sample, all FoPLs significantly outperformed the RIs. However, as before, the magnitude of the effect differed according to FoPL. The Nutri-Score was associated with the highest improvement in ability to correctly rank product healthiness (Odds Ratio [95% confidence interval]: OR = 3.07 [2.75–3.43], *p*-value < 0.0001), followed by the MTL (OR = 1.77 [1.59–1.98], *p*-value < 0.0001), the HSR (OR = 1.37 [1.23–1.53], *p*-value < 0.0001), and the Warning symbol (OR = 1.28 [1.15–1.43], *p*-value < 0.0001). Furthermore, the Nutri-Score performed the best in all 12 countries, with ORs ranging from 2.14 [1.48–3.10] (*p*-value = 0.001) in Argentina to 4.45 [3.02–6.56] (*p*-value < 0.0001) in Singapore. The results for the remaining FoPLs were heterogeneous across countries; however, in most instances the MTL was the second-best performing label after the Nutri-Score. The HSR and the Warning symbol also significantly outperformed the RIs in most countries, but the effects were weaker. Similar trends were found when analyses were performed separately for each food category, with FoPLs appearing somewhat more effective in the cake products category compared with the other two categories (Table S1).

In sensitivity analyses including only participants who recalled seeing the FoPL during the survey, higher magnitudes of effects were observed, and the order of FoPLs according to improvement in participants’ ability to correctly rank the nutritional quality of food products was slightly modified (Table S2). In the full sample for all food categories, the Nutri-Score performed best compared to the RIs (OR = 3.64 [3.20–4.14], *p*-value < 0.0001), followed by the Warning symbol (OR = 2.00 [1.74–2.31], *p*-value < 0.0001), the MTL (OR = 1.87 [1.65–2.12], *p*-value < 0.0001), and the HSR (OR = 1.76 [1.54–2.02], *p*-value < 0.0001). Similar trends were observed across countries. 

## 4. Discussion

In the present study, all five FoPLs significantly improved the ability of individuals to rank products according to their nutritional quality, but with notable differences across FoPL types. Compared to the RIs, which emerged as the least effective FoPL, the Nutri-Score produced the highest improvement in ranking ability, followed by the MTL, HSR, and Warning symbol. Similar trends were observed across all three food categories and all 12 countries. However, the insignificant results in individual countries may be partly explained by multiple testing corrections and lack of sufficient statistical power for some of the models. 

The fact that all FoPLs were associated with a significant improvement in food healthfulness ranking ability compared to a no label situation is consistent with the literature, suggesting that FoPLs can help consumers discriminate between the nutritional quality of different food products and identify healthier food choices [25,26,27]. In addition, the interpretive FoPLs (Nutri-Score, MTL, HSR, and Warning symbol) significantly outperformed the non-interpretive label (RIs), which is in line with the results of prior studies [31,40,42]. The comparatively weak performance of the RIs may be explained in particular by its reliance on numeric information (grams and percentages), and its evaluation per portion [30,43,44]. Nutrient-specific labels providing only numerical information have been consistently found to be poorly understood by consumers, in particular by those with low educational levels, as they entail a high cognitive workload to interpret [26,27,28,30,31,41,45]. However, even though interpretive labels clearly outperform non-interpretive ones, design features are also likely to result in varying degrees of FoPL effectiveness. Hence, it appears important to better understand the characteristics of interpretive FoPLs that improve consumers’ understanding of the nutritional value of foods. 

Given the findings of the present study, two major features appear to influence FoPL understanding: use of colours and summary versus nutrient-specific information. Interpretive FoPLs associated with the highest increase in objective understanding were the Nutri-Score and the MTL, which were the only two colour-coded labels among the five FoPLs tested. It has been demonstrated that the use of colours is key regarding FoPL salience, as colours tend to capture attention [27,31,43,46,47,48,49,50]. Moreover, the use of the well-known polychromatic green-red scale might be an important feature of FoPL colour coding. Indeed, green and red colours, corresponding to recognised signals, may be easier to understand and interpret, with green being associated with safety and a “go” signal, and red being associated with danger and a “stop” signal [33,51]. Thus, the presence of a colour-coded FoPL may be effective at different stages of information processing: at an early stage by drawing attention to the label and at a later stage by aiding understanding [50]. In contrast, the HSR and the Warning symbol, which are monochromatic labels, were the two interpretive FoPLs with the lowest percentage of participants recalling seeing the label during the survey and the weakest performance regarding objective understanding. In sensitivity analyses, when considering only participants recalling seeing the FoPL, the results for the Warning symbol were substantially improved. This suggests that this type of nutrition label is well understood once identified and might even result in improved effectiveness if presented in more salient colours [49]. 

The other key element of an FoPL’s format that may influence its ability to increase understanding of nutrition quality is the presence of a summary indicator rather than merely nutrient-specific information. Indeed, among the colour-coded FoPLs tested in the study, the Nutri-Score summary label performed notably better than the nutrient-specific MTL. This finding is consistent with prior studies’ findings that summary indicators are more easily understood by consumers [27,31,40] and limit potential confusion related to the interpretation of nutritional terms (e.g., saturated fats, sugars, and sodium) [52]. These FoPLs provide synthesised information that may be associated with a reduced cognitive workload, resulting in faster processing and less difficulty in understanding the meaning of the information provided [30,35]. While the MTL provides five different pieces of information on specific nutrients, the Nutri-Score summarises the overall nutritional quality of the product. Generally, these types of nutrition labels appear to be more efficient and useful tools with which to influence consumers’ choices at the point-of-purchase where decisions are made in a very short time period [40]. Hence, the stronger performance of the Nutri-Score regarding objective understanding may be related to its use of the combination of both semantic colours and a simple and intuitive summary graded design. 

In the present study, similar patterns of consumers’ objective understanding of the FoPLs were observed across the 12 countries, with comparable magnitudes of effects, even if the geographical area and food cultural background are quite different. More specifically, the Nutri-Score showed greater effectiveness compared with the other four FoPLs, even in countries where an alternative official FoPL is already implemented. That was notably the case in the UK, where the MTL was introduced on pre-packed foods in 2004, and Australia, where the HSR system has been applied on food packages since 2014. In these two countries, the Nutri-Score performed better than the MTL and the HSR, respectively, suggesting that its graphical assets may outweigh any potential benefits of familiarity. This finding is consistent with the results of a study that compared evaluation, use, intentions, and product choices among three nutrition labels in two countries with different FoPL histories [53]. The authors observed that familiarity with a FoPL influenced self-reported evaluations and use intentions only, but all FoPLs were equally effective in encouraging healthier food choices. This homogeneous result across countries may be partly explained by the fact that these key elements of interpretations and, more specifically, the use of colour-coding with the green-red polychromatic scale are internationally understood. Indeed, given the specific neurobiological aspects of color recognition in humans, green/red cues are considered to be the most easily differentiated colors [54]. In the present study, very few disparities were found among countries, with only a small number of instances in which specific FoPLs were more strongly associated with objective understanding in some but not other countries. For example, the HSR effect was significant in Australia, Bulgaria, and Singapore only, and the Warning symbol was significant in Singapore only compared to the RIs. These limited instances of discrepancies in understanding and use of FoPLs among countries may be partly attributed to the local context and the impact and strength of the public discourse on nutrition and labelling [41]. 

Strengths of this study included the large sample size and the recruitment of participants in 12 countries from different continents (Europe, North and South America, Asia, and Oceania) that facilitated cross-cultural comparisons of FoPL effects. In addition, the use of sets of three food products (rather than evaluation of sets of two as is often done in other studies) approximated realistic situations while decreasing the risk of correct responses simply by chance. Furthermore, the stimuli were developed to ensure a clear nutritional difference between the products were communicated by the information provided by each FOPL to facilitate the ranking process. However, these methodological choices led to the exclusion of endorsement schemes from the test as understanding of these FoPLs is difficult to assess across more than two products at once (e.g., no discrimination would be possible between two products without any endorsement labels on their packages). Finally, a potential learning effect was also controlled for through the randomisation of the order of presentation within the sets and across food categories. 

However, some limitations of the study should be acknowledged. A primary limitation was the use of a web panel using set quotas across countries rather than attempting to generate population representative samples. Thus, caution is required regarding extrapolation of the results. However, participants in all 12 countries were recruited using the same methods and criteria. Second, results may have been influenced by the familiarity in the cases where one of the five FoPLs was already implemented in a particular country. However, this was taken into account by adjusting the country of origin in the analyses including the full sample. Third, participants did not have access to the nutritional composition of the products used in the study, which differs from real-life situations in which consumers would often be able to access more detailed nutrition information on the back of the pack. This might have led to fewer correct responses in the no label situation than in real life settings. However, it has been demonstrated that back-of-package information is rarely considered when grocery shopping [54]. Finally, the study was conducted as an online experiment and not in a real shopping situation, in which many additional factors are likely influence consumers’ perceptions and choices. Indeed, time pressure and the familiarity of consumers with specific food products and brands may influence purchasing choices, while the timing of the questionnaire completion in the present study was not limited, and fictional foods were used.

## 5. Conclusions

In conclusion, though all FoPLs tested in this study improved consumers’ understanding of the nutritional quality of food products, their performance varied, and the combination of colour-coded information with a summary graded graphical design appeared as the most effective. Indeed, among the tested labels, Nutri-Score emerged as the most efficient FoPL in conveying information on the nutritional quality of foods and thus helping consumers to discriminate between products. Moreover, it appeared to be clearly understood in diverse sociocultural contexts and even outweighed potential familiarity of consumers with other labels. Policy-makers should be encouraged to conduct comparative studies including such an alternative to ensure that they implement the most efficient scheme.

## Figures and Tables

**Table 1:** Individual characteristics of the study sample (*n* = 12,015).

	Argentina	Australia	Bulgaria	Canada	Denmark	France	Germany	Mexico	Singapore	Spain	USA	UK	Total
	*n* (%)	*n* (%)	*n* (%)	*n* (%)	*n* (%)	*n* (%)	*n* (%)	*n* (%)	*n* (%)	*n* (%)	*n* (%)	*n* (%)	*n* (%)
**Sex**													
Men	496 (49.55)	500 (50.00)	508 (50.15)	500 (50.00)	500 (50.00)	500 (50.00)	500 (50.00)	501 (50.05)	500 (50.00)	500 (50.00)	500 (50.00)	500 (50.00)	6005 (49.98)
Women	505 (50.45)	500 (50.00)	505 (49.85)	500 (50.00)	500 (50.00)	500 (50.00)	500 (50.00)	500 (49.95)	500 (50.00)	500 (50.00)	500 (50.00)	500 (50.00)	6010 (50.02)
**Age, years**													
18–30	336 (33.57)	331 (33.10)	359 (35.44)	332 (33.20)	328 (32.80)	333 (33.30)	340 (34.00)	340 (33.97)	340 (34.00)	339 (33.90)	332 (33.20)	332 (33.20)	4042 (33.64)
31–50	332 (33.17)	335 (33.50)	379 (37.41)	334 (33.40)	333 (33.30)	333 (33.30)	330 (33.00)	335 (33.47)	337 (33.70)	331 (33.10)	334 (33.40)	334 (33.40)	4047 (33.68)
>50	333 (33.27)	334 (33.40)	275 (27.15)	334 (33.40)	339 (33.90)	334 (33.40)	330 (33.00)	326 (32.57)	323 (32.30)	330 (33.00)	334 (33.40)	334 (33.40)	3926 (32.68)
**Educational level**													
Primary education	14 (1.40)	9 (0.90)	6 (0.59)	26 (2.60)	94 (9.40)	17 (1.70)	97 (9.70)	2 (0.20)	6 (0.60)	21 (2.10)	136 (13.60)	7 (0.70)	435 (3.62)
Secondary education	256 (25.57)	263 (26.30)	142 (14.02)	263 (26.30)	172 (17.20)	183 (18.30)	382 (38.20)	102 (10.19)	123 (12.30)	316 (31.60)	232 (23.20)	381 (38.10)	2815 (23.43)
Trade certificate	244 (24.38)	196 (19.60)	252 (24.88)	203 (20.30)	391 (39.10)	266 (26.60)	241 (24.10)	145 (14.49)	204 (20.40)	166 (16.60)	115 (11.50)	144 (14.40)	2567 (21.36)
University undergraduate degree	372 (37.16)	389 (38.90)	262 (25.86)	358 (35.80)	210 (21.00)	334 (33.40)	129 (12.90)	544 (54.35)	494 (49.40)	282 (28.20)	349 (34.90)	343 (34.30)	4066 (33.84)
University postgraduate degree	115 (11.49)	143 (14.30)	351 (34.65)	150 (15.00)	133 (13.30)	200 (20.00)	151 (15.10)	208 (20.78)	173 (17.30)	215 (21.50)	168 (16.80)	125 (12.50)	2132 (17.74)
**Level of income**													
High	330 (32.97)	335 (33.50)	370 (36.53)	325 (32.50)	320 (32.00)	334 (33.40)	327 (32.70)	331 (33.07)	324 (32.40)	330 (33.00)	325 (32.50)	335 (33.50)	3986 (33.18)
Medium	333 (33.27)	334 (33.40)	359 (35.44)	335 (33.50)	340 (34.00)	333 (33.30)	333 (33.30)	330 (32.97)	336 (33.60)	330 (33.00)	335 (33.50)	335 (33.50)	4033 (33.57)
Low	338 (33.77)	331 (33.10)	284 (28.04)	340 (34.00)	340 (34.00)	333 (33.30)	340 (34.00)	340 (33.97)	340 (34.00)	340 (34.00)	340 (34.00)	330 (33.00)	3996 (33.26)
**Responsible for grocery shopping**													
Yes	809 (80.82)	719 (71.90)	599 (59.13)	750 (75.00)	690 (69.00)	863 (86.30)	769 (76.90)	819 (81.82)	638 (63.80)	747 (74.70)	793 (79.30)	750 (75.0)	8946 (74.46)
No	45 (4.50)	74 (7.40)	64 (6.32)	45 (4.50)	55 (5.50)	21 (2.10)	31 (3.10)	34 (3.40)	81 (8.10)	35 (3.50)	56 (5.60)	35 (3.50)	576 (4.79)
Share job equally	147 (14.69)	207 (20.70)	350 (34.55)	205 (20.50)	255 (25.50)	116 (11.60)	200 (20.00)	148 (14.79)	281 (28.10)	218 (21.80)	151 (15.10)	215 (21.50)	2493 (20.75)
**Self-estimated diet quality**													
I eat a very unhealthy diet	17 (1.70)	4 (0.40)	48 (4.74)	19 (1.90)	12 (1.20)	20 (2.00)	34 (3.40)	16 (1.60)	11 (1.10)	11 (1.10)	28 (2.80)	11 (1.10)	231 (1.92)
I eat a mostly unhealthy diet	227 (22.68)	159 (15.90)	609 (60.12)	171 (17.10)	199 (19.90)	182 (18.20)	202 (20.20)	274 (27.37)	220 (22.00)	162 (16.20)	217 (21.70)	211 (21.10)	2833 (23.58)
I eat a mostly healthy diet	603 (60.24)	763 (76.30)	341 (33.66)	729 (72.90)	727 (72.70)	660 (66.00)	677 (67.70)	547 (54.65)	691 (69.10)	711 (71.10)	638 (63.80)	715 (71.50)	7802 (64.94)
I eat a very healthy diet	154 (15.38)	74 (7.40)	15 (1.48)	81 (8.10)	62 (6.20)	138 (13.80)	87 (8.70)	164 (16.38)	78 (7.80)	116 (11.60)	117 (11.70)	63 (6.30)	1149 (9.56)
**Nutrition knowledge**													
I do not know anything about nutrition	18 (1.80)	7 (0.70)	9 (0.89)	10 (1.00)	10 (1.00)	51 (5.10)	15 (1.50)	14 (1.40)	5 (0.50)	26 (2.60)	16 (1.60)	17 (1.70)	198 (1.65)
I am not very knowledgeable about nutrition	244 (24.38)	174 (17.40)	210 (20.73)	141 (14.10)	166 (16.60)	408 (40.80)	193 (19.30)	289 (28.87)	198 (19.80)	287 (28.70)	147 (14.70)	235 (23.50)	2692 (22.41)
I am somewhat knowledgeable about nutrition	557 (55.64)	695 (69.50)	627 (61.9)	658 (65.80)	638 (63.80)	380 (38.00)	617 (61.70)	554 (55.34)	664 (66.40)	609 (60.90)	641 (64.10)	664 (66.40)	7304 (60.79)
I am very knowledgeable about nutrition	182 (18.18)	124 (12.40)	167 (16.49)	191 (19.10)	186 (18.60)	161 (16.10)	175 (17.50)	144 (14.39)	133 (13.30)	78 (7.80)	196 (19.60)	84 (8.40)	1821 (15.16)
**Did you see the FoP label during the survey?**													
No	165 (16.48)	168 (16.80)	311 (30.70)	242 (24.20)	351 (35.10)	321 (32.10)	306 (30.60)	176 (17.58)	246 (24.60)	275 (27.50)	240 (24.00)	256 (25.60)	3057 (25.44)
Unsure	109 (10.89)	47 (4.70)	139 (13.72)	83 (8.30)	75 (7.50)	75 (7.50)	140 (14.00)	94 (9.39)	129 (12.90)	150 (15.00)	77 (7.70)	90 (9.00)	1208 (10.05)
Yes	727 (72.63)	508 (50.80)	563 (55.58)	675 (67.50)	574 (57.40)	604 (60.40)	554 (55.40)	731 (73.03)	625 (62.50)	575 (57.50)	683 (68.30)	654 (65.40)	7473 (62.20)
**Participants who recalled seeing the FoPL they were exposed to**													
HSR	135 (67.50)	112 (77.78)	85 (42.08)	127 (63.50)	105 (52.50)	103 (51.50)	90 (45.00)	133 (66.17)	109 (54.50)	82 (41.00)	137 (68.50)	109 (54.50)	1327 (56.54)
MTL	161 (80.50)	102 (70.34)	120 (59.11)	145 (72.50)	125 (62.50)	138 (69.00)	128 (64.00)	170 (85.00)	147 (73.50)	140 (70.00)	151 (75.50)	160 (80.00)	1687 (71.85)
Nutri-Score	142 (71.00)	99 (68.75)	152 (75.25)	144 (72.00)	131 (65.50)	130 (65.00)	136 (68.00)	152 (76.00)	125 (62.50)	107 (53.50)	155 (77.50)	138 (69.00)	1611 (68.67)
RIs	163 (81.09)	120 (82.76)	112 (55.17)	149 (74.87)	133 (66.50)	131 (65.50)	128 (64.00)	165 (82.50)	152 (76.00)	155 (77.50)	150 (75.00)	153 (76.50)	1711 (72.87)
Warning symbol	126 (63.00)	75 (51.72)	94 (46.31)	110 (54.73)	80 (40.00)	102 (51.00)	72 (36.00)	111 (55.50)	92 (46.00)	91 (45.50)	90 (45.00)	94 (47.00)	1137 (48.40)

HSR: Health Star Rating system; MTL: Multiple Traffic Lights; RIs: Reference Intake.

**Table 2:** Associations ^a^ between FoPLs and change in ability to correctly rank products between no label and labelling conditions.

Countries	*n*	HSR		MTL		Nutri-Score		Warning Symbol	
		OR [95% CI]	*p*	OR [95% CI]	*p*	OR [95% CI]	*p*	OR [95% CI]	*p*
All countries	12,015	1.37 [1.23–1.53]	**<0.0001**	1.77 [1.59–1.98]	**<0.0001**	3.07 [2.75–3.43]	**<0.0001**	1.28 [1.15–1.43]	**<0.0001**
Argentina	1001	1.14 [0.79–1.66]	0.7	1.22 [0.84–1.78]	0.6	2.14 [1.48–3.10]	**0.001**	0.98 [0.67–1.43]	1.0
Australia	1000	1.86 [1.27–2.74]	**0.02**	1.52 [1.03–2.24]	0.2	4.15 [2.82–6.11]	**<0.0001**	1.41 [0.95–2.08]	0.3
Bulgaria	1013	1.97 [1.31–2.97]	**0.01**	1.12 [0.74–1.67]	0.8	2.34 [1.55–3.53]	**0.001**	1.28 [0.85–1.91]	0.6
Canada	1000	1.49 [1.02–2.17]	0.2	1.71 [1.17–2.49]	**0.05**	3.30 [2.27–4.80]	**<0.0001**	1.35 [0.92–1.97]	0.4
Denmark	1000	1.09 [0.75–1.60]	0.8	1.65 [1.13–2.40]	0.09	2.46 [1.69–3.58]	**<0.0001**	1.02 [0.70–1.49]	1.0
France	1000	1.53 [1.03–2.27]	0.2	2.42 [1.63–3.57]	**0.0002**	4.29 [2.90–6.35]	**<0.0001**	1.51 [1.02–2.24]	0.2
Germany	1000	1.20 [0.80–1.80]	0.6	2.15 [1.44–3.21]	**0.003**	2.72 [1.83–4.05]	**<0.0001**	1.10 [0.73–1.65]	0.8
Mexico	1001	1.30 [0.89–1.90]	0.5	2.61 [1.78–3.81]	**<0.0001**	2.67 [1.83–3.90]	**<0.0001**	1.63 [1.11–2.39]	0.1
Singapore	1000	1.99 [1.35–2.93]	**0.007**	2.06 [1.40–3.03]	**0.004**	4.45 [3.02–6.56]	**<0.0001**	2.04 [1.39–3.00]	**0.005**
Spain	1000	0.81 [0.55–1.20]	0.6	1.77 [1.20–2.61]	**0.04**	3.00 [2.04–4.41]	**<0.0001**	1.17 [0.79–1.72]	0.7
USA	1000	1.28 [0.87–1.87]	0.5	1.96 [1.34–2.86]	**0.007**	3.10 [2.12–4.53]	**<0.0001**	1.06 [0.72–1.56]	0.9
UK	1000	1.32 [0.89–1.95]	0.5	1.97 [1.34–2.89]	**0.008**	4.21 [2.86–6.20]	**<0.0001**	1.25 [0.85–1.85]	0.6

^a^ The reference of the multivariate ordinal logistic regression for the categorical variable “label” was the Reference Intakes. The multivariate model was adjusted according to sex, age, educational level, level of income, responsibility for grocery shopping, self-estimated diet quality, and self-estimated nutrition knowledge level. HSR: Health Star Rating system; MTL: Multiple Traffic Lights; OR: Odds Ratio; CI: Confidence Interval. Bold values correspond to significant results corrected for multiple testing (*p*-value ≤ 0.05).

## Supplementary Materials

The following are available online at http://www.mdpi.com/2072-6643/10/10/1542/s1, Figure S1: Example of the set of three cakes tested in the present study with the associated FoPLs; Figure S2: Example of the set of three breakfast cereals tested in the present study with the associated FoPLs; Table S1: Associations between FoPLs and the improvement in the ability to correctly rank products between no label and labelling conditions, by food category; Table S2: Associations between FoPLs and change in ability to correctly rank products between no label and labelling conditions among participants who reported seeing the label during the survey.
