# Supplementary material for: Objective Understanding of Front-of-Package Nutrition Labels: An International Comparative Experimental Study across 12 Countries
Source: Nutrients. 2018 Oct 18;10(10):1542. doi: 10.3390/nu10101542 (PMC6213801; doi:10.3390/nu10101542)
Supplement: Supplementary file 1 [file nutrients-10-01542-s001.pdf]

<table>
<tr><th>Labelling condition</th><th>Example of one food category : Cakes</th></tr>
<tr><td>No label</td><td>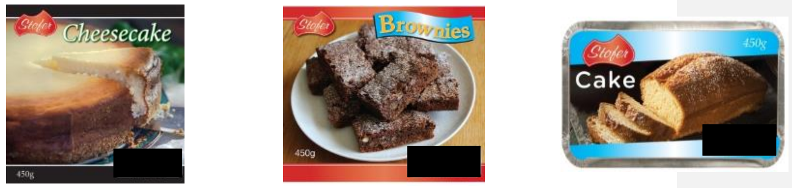
</td></tr>
<tr><td>Health Star Rating system</td><td>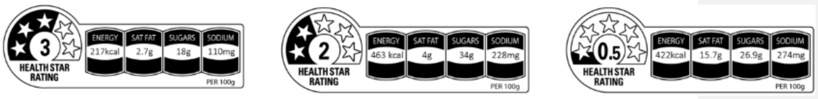
</td></tr>
<tr><td>Multiple Traffic Lights</td><td>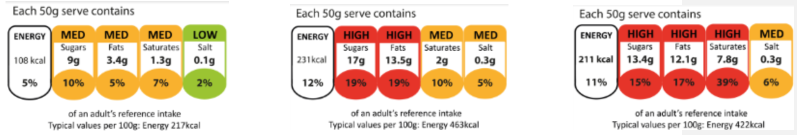
</td></tr>
<tr><td>Nutri-Score</td><td>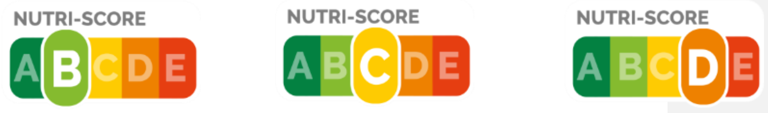
</td></tr>
<tr><td>Reference Intakes label</td><td>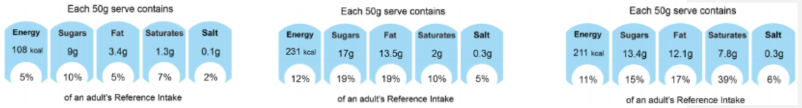
</td></tr>
<tr><td>Warning symbol</td><td>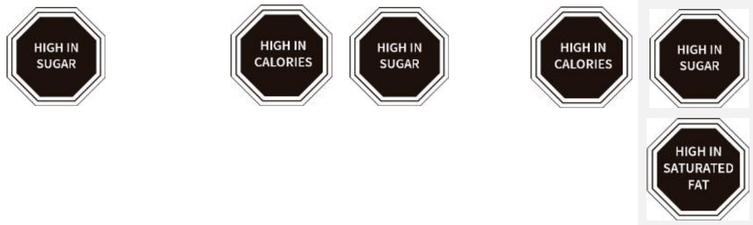
</td></tr>
</table>

Figure S1. Example of the set of three cakes tested in the present study with the associated FoPLs.

Labelling condition	Example of one food category : Breakfast cereals
No label	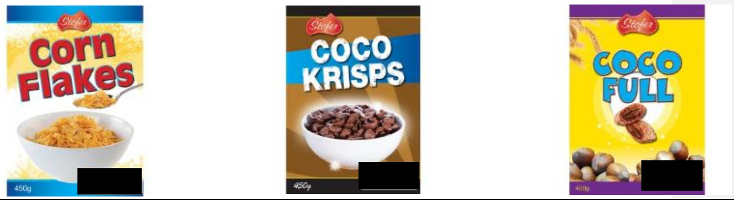
Health Star Rating system	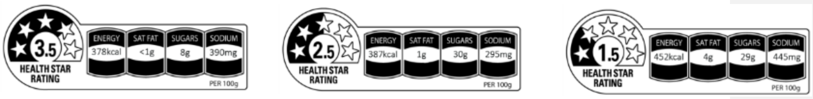
Multiple Traffic Lights	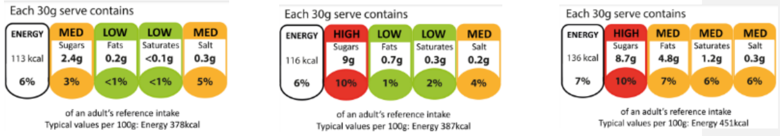
Nutri-Score	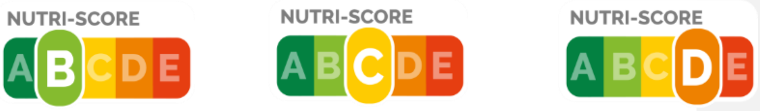
Reference Intakes label	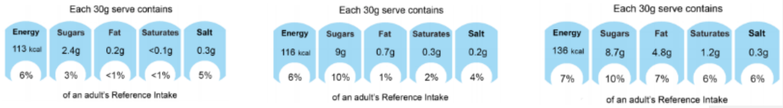
Warning symbol	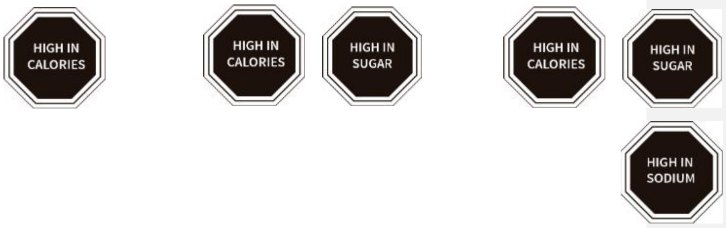

Figure S2. Example of the set of three breakfast cereals tested in the present study with the associated FoPLs.

Table S1. Associations[a] between FoPLs and the improvement in the ability to correctly rank products between no label and labelling conditions, by food category

Countries	N	HSR		MTL		Nutri-Score		Warning symbol	
		OR (95% CI)	P	OR (95% CI)	P	OR (95% CI)	P	OR (95% CI)	P
**Pizza**									
All countries	11652	1.18 [1.03-1.36]	0.06	1.46 [1.28-1.68]	**<.0001**	2.04 [1.79-2.34]	**<.0001**	1.01 [0.88-1.16]	0.9
Argentina	991	1.15 [0.74-1.78]	0.9	1.13 [0.73-1.75]	0.9	1.56 [1.01-2.40]	0.3	0.85 [0.55-1.33]	0.8
Australia	962	1.50 [0.92-2.45]	0.5	1.48 [0.90-2.43]	0.5	2.22 [1.37-3.59]	**0.04**	0.81 [0.49-1.36]	0.8
Bulgaria	960	1.77 [1.08-2.88]	0.3	0.95 [0.59-1.54]	1.0	2.34 [1.43-3.82]	**0.03**	1.35 [0.83-2.19]	0.6
Canada	990	0.88 [0.56-1.39]	0.9	1.06 [0.68-1.67]	1.0	1.63 [1.05-2.52]	0.3	0.79 [0.50-1.25]	0.7
Denmark	905	0.83 [0.50-1.37]	0.8	1.29 [0.79-2.13]	0.7	1.31 [0.80-2.14]	0.7	0.78 [0.47-1.29]	0.7
France	979	1.77 [1.08-2.91]	0.3	1.97 [1.20-3.25]	0.2	2.71 [1.65-4.43]	**0.006**	1.82 [1.11-3.01]	0.3
Germany	979	0.96 [0.57-1.59]	1.0	1.45 [0.88-2.37]	0.6	1.84 [1.13-3.01]	0.3	0.94 [0.56-1.57]	1.0
Mexico	997	1.03 [0.65-1.65]	1.0	1.66 [1.05-2.64]	0.3	1.61 [1.02-2.55]	0.3	0.85 [0.53-1.36]	0.8
Singapore	970	1.71 [1.05-2.78]	0.3	1.52 [0.93-2.46]	0.5	3.14 [1.95-5.08]	**0.0007**	1.51 [0.93-2.45]	0.5
Spain	994	0.73 [0.43-1.22]	0.6	1.68 [1.03-2.73]	0.3	2.11 [1.30-3.42]	0.08	0.72 [0.43-1.20]	0.6
USA	985	1.20 [0.75-1.91]	0.8	2.02 [1.27-3.21]	0.08	2.64 [1.67-4.17]	**0.004**	1.09 [0.68-1.75]	0.9
UK	940	1.21 [0.72-2.04]	0.8	1.87 [1.12-3.10]	0.3	2.41 [1.46-3.98]	**0.03**	1.05 [0.63-1.76]	1.0
**Cakes**									
All countries	11705	1.84 [1.59-2.12]	**<.0001**	2.70 [2.34-3.11]	**<.0001**	4.62 [4.02-5.31]	**<.0001**	1.89 [1.64-2.18]	**<.0001**
Argentina	977	1.12 [0.70-1.80]	0.8	1.44 [0.91-2.30]	0.4	2.44 [1.55-3.83]	**0.001**	1.07 [0.67-1.72]	0.9
Australia	940	2.11 [1.29-3.42]	**0.02**	1.79 [1.10-2.91]	0.1	5.94 [3.68-9.57]	**<.0001**	2.08 [1.28-3.39]	**0.03**
Bulgaria	1003	2.94 [1.68-5.15]	**0.002**	1.51 [0.87-2.63]	0.4	2.93 [1.67-5.13]	**0.002**	1.63 [0.94-2.84]	0.3
Canada	949	2.13 [1.32-3.45]	**0.02**	2.81 [1.74-4.52]	**0.0003**	4.29 [2.68-6.85]	**<.0001**	1.84 [1.14-3.00]	0.09
Denmark	964	1.68 [1.04-2.71]	0.2	2.82 [1.75-4.56]	**0.0003**	4.26 [2.66-6.82]	**<.0001**	1.62 [1.00-2.61]	0.2
France	996	2.04 [1.19-3.49]	0.06	4.90 [2.92-8.22]	**<.0001**	9.03 [5.4-15.11]	**<.0001**	2.18 [1.27-3.74]	**0.03**
Germany	976	2.01 [1.14-3.53]	0.1	4.12 [2.38-7.15]	**<.0001**	5.37 [3.11-9.28]	**<.0001**	2.18 [1.23-3.87]	0.05
Mexico	995	2.53 [1.48-4.33]	**0.007**	5.67 [3.35-9.60]	**<.0001**	5.49 [3.25-9.27]	**<.0001**	4.07 [2.39-6.92]	**<.0001**
Singapore	996	2.58 [1.59-4.16]	**0.001**	3.01 [1.87-4.84]	**0.0001**	5.78 [3.60-9.26]	**<.0001**	2.36 [1.46-3.82]	**0.005**
Spain	975	1.14 [0.68-1.91]	0.8	3.09 [1.87-5.11]	**0.0002**	4.72 [2.88-7.75]	**<.0001**	2.10 [1.26-3.48]	**0.03**
USA	954	1.32 [0.78-2.22]	0.6	2.32 [1.41-3.83]	**0.009**	3.53 [2.16-5.77]	**<.0001**	1.24 [0.74-2.09]	0.7
UK	980	1.87 [1.10-3.17]	0.1	2.64 [1.57-4.44]	**0.003**	7.53 [4.55-12.46]	**<.0001**	2.18 [1.29-3.68]	**0.03**
**Breakfast cereals**									
All countries	11509	1.30 [1.13-1.49]	**0.001**	1.39 [1.21-1.59]	**<.0001**	2.41 [2.11-2.76]	**<.0001**	1.14 [1.00-1.31]	0.1
Argentina	973	1.64 [1.05-2.56]	0.3	1.28 [0.82-2.01]	0.7	2.55 [1.64-3.96]	**0.001**	1.06 [0.68-1.67]	1.0
Australia	969	1.85 [1.15-2.97]	0.2	1.39 [0.86-2.26]	0.7	3.24 [2.03-5.17]	**0.0001**	1.68 [1.04-2.72]	0.3
Bulgaria	964	1.50 [0.80-2.80]	0.7	1.35 [0.72-2.53]	0.8	2.02 [1.09-3.75]	0.3	0.94 [0.50-1.78]	1.0
Canada	983	1.79 [1.13-2.85]	0.2	1.75 [1.10-2.78]	0.2	3.60 [2.29-5.65]	**<.0001**	1.84 [1.16-2.93]	0.1
Denmark	978	0.90 [0.56-1.45]	0.9	1.11 [0.70-1.77]	0.9	1.66 [1.05-2.62]	0.3	0.76 [0.47-1.23]	0.7
France	892	0.88 [0.53-1.46]	0.9	1.19 [0.72-1.95]	0.9	1.93 [1.19-3.15]	0.1	0.89 [0.54-1.47]	0.9
Germany	879	1.45 [0.83-2.52]	0.7	1.68 [0.98-2.90]	0.4	2.55 [1.49-4.34]	**0.02**	0.95 [0.54-1.68]	1.0
Mexico	994	1.19 [0.76-1.86]	0.8	1.74 [1.12-2.69]	0.2	2.06 [1.33-3.18]	**0.03**	1.29 [0.83-2.02]	0.7
Singapore	974	1.51 [0.93-2.47]	0.5	1.32 [0.81-2.16]	0.7	2.85 [1.77-4.59]	**0.0008**	1.65 [1.02-2.69]	0.3
Spain	940	0.64 [0.39-1.06]	0.4	0.95 [0.58-1.55]	1.0	1.98 [1.25-3.14]	0.07	0.91 [0.56-1.48]	1.0
USA	980	1.36 [0.86-2.17]	0.7	1.61 [1.02-2.54]	0.3	2.79 [1.77-4.38]	**0.0007**	1.22 [0.77-1.95]	0.8
UK	983	1.30 [0.81-2.10]	0.7	1.62 [1.02-2.58]	0.3	2.74 [1.74-4.33]	**0.0008**	0.92 [0.57-1.50]	1.0

[a] The reference of the multivariate ordinal logistic regression was the Reference Intakes.
The multivariate model was adjusted on sex, age, educational level, level of income, responsibility for grocery shopping, self-estimated diet quality, and self-estimated nutrition knowledge level.
HSR: Health Star Rating system; MTL: Multiple Traffic Lights; OR: Odds Ratio; CI: Confidence Interval.
Bold values correspond to significant results corrected for multiple testing (p-value≤0.05).

Deleted: s

Table S2. Associations[a] between FoPLs and change in ability to correctly rank products between no label and labelling conditions among participants who reported seeing the label during the survey

Countries	N	HSR		MTL		Nutri-Score		Warning symbol	
		OR [95% CI]	P	OR [95% CI]	P	OR [95% CI]	P	OR [95% CI]	P
All countries	7473	1.76 [1.54-2.02]	**<.0001**	1.87 [1.65-2.12]	**<.0001**	3.64 [3.20-4.14]	**<.0001**	2.00 [1.74-2.31]	**<.0001**
Argentina	727	1.56 [1.01-2.39]	0.2	1.44 [0.96-2.17]	0.3	2.72 [1.78-4.15]	**<.0001**	1.33 [0.85-2.06]	0.5
Australia	508[b]	1.63 [0.99-2.70]	0.2	1.59 [0.95-2.67]	0.3	6.49 [3.81-11.07]	**<.0001**	2.89 [1.66-5.04]	**0.002**
Bulgaria	563	2.71 [1.54-4.77]	**0.006**	1.11 [0.67-1.85]	0.8	2.99 [1.82-4.90]	**0.0003**	1.11 [0.65-1.91]	0.8
Canada	675	2.11 [1.35-3.30]	**0.01**	1.87 [1.22-2.87]	**0.03**	4.00 [2.58-6.18]	**<.0001**	2.44 [1.53-3.87]	**0.002**
Denmark	574	1.72 [1.06-2.80]	0.1	2.10 [1.32-3.34]	**0.02**	3.39 [2.13-5.39]	**<.0001**	1.38 [0.81-2.35]	0.5
France	604	1.93 [1.17-3.20]	0.07	2.43 [1.53-3.87]	**0.002**	5.46 [3.39-8.79]	**<.0001**	2.67 [1.61-4.42]	**0.002**
Germany	554	1.57 [0.92-2.67]	0.3	2.24 [1.38-3.63]	**0.01**	2.86 [1.77-4.60]	**0.0003**	2.72 [1.55-4.77]	**0.006**
Mexico	731	1.62 [1.05-2.51]	0.1	2.69 [1.78-4.05]	**<.0001**	2.64 [1.73-4.02]	**0.0001**	2.85 [1.80-4.52]	**0.0002**
Singapore	625	2.88 [1.79-4.64]	**0.0003**	2.02 [1.30-3.13]	**0.02**	4.74 [2.99-7.52]	**<.0001**	2.92 [1.78-4.79]	**0.0003**
Spain	575	1.15 [0.68-1.95]	0.8	2.26 [1.44-3.55]	**0.005**	4.21 [2.60-6.83]	**<.0001**	2.38 [1.44-3.94]	**0.008**
USA	683	1.45 [0.94-2.24]	0.3	1.74 [1.14-2.66]	0.07	3.64 [2.38-5.58]	**<.0001**	1.79 [1.10-2.93]	0.1
UK	654	1.73 [1.07-2.78]	0.1	1.81 [1.18-2.78]	**0.05**	5.83 [3.71-9.16]	**<.0001**	2.04 [1.24-3.34]	**0.04**

[a] The reference of the multivariate ordinal logistic regression was the Reference Intakes.
The multivariate model was adjusted on sex, age, educational level, level of income, responsibility for grocery shopping, self-estimated diet quality, and self-estimated nutrition knowledge level.
[b] 277 participants from Australia did not have access to the question “Did you see this FOP label during the survey ?”, for technical issues.
HSR: Health Star Rating system; MTL: Multiple Traffic Lights; OR: Odds Ratio; CI: Confidence Interval.
Bold values correspond to significant results corrected for multiple testing (p-value≤0.05).
